# Prevalence and characteristics of hepatitis C virus infection in Shenyang City, Northeast China, and prediction of HCV RNA positivity according to serum anti-HCV level: retrospective review of hospital data

**DOI:** 10.1186/s12985-020-01316-y

**Published:** 2020-03-16

**Authors:** Yurong Li, Lianrong Zhao, Nan Geng, Weijia Zhu, Hongbo Liu, Han Bai

**Affiliations:** 1 Department of Infectious Diseases, Shengjing Hospital of China Medical University, Shenyang, Liaoning P.R. China 110004; 2 Department of Health Statistics, School of Public Health, China Medical University, Shenyang, Liaoning P.R. China 110122

**Keywords:** Hepatitis C virus, Anti-HCV, HCV RNA, Epidemiology, Correlation

## Abstract

**Objective:**

The prevalence of hepatitis C virus (HCV) infection is typically evaluated based on the current rate of positivity of anti-HCV antibody; however, HCV RNA positivity is considered the main criterion for antiviral treatment of HCV infection in the clinical setting. In this study, we evaluated the prevalence of HCV infection based on anti-HCV and HCV RNA detection in the population of Liaoning Province, and investigated the correlation between serum HCV RNA positivity and anti-HCV levels.

**Methods:**

A total of 192,202 patients who underwent serum anti-HCV examination at Shengjing Hospital in 2018 were enrolled in the study. Anti-HCV production was tested using a chemiluminescence assay, and serum HCV RNA detection was performed with Roche COBAS TaqMan (CTM) Analyzer.

**Results:**

The prevalence of anti-HCV was 1.21 and 0.93% among male and female patients in Liaoning Province, respectively. The positive rates of anti-HCV and serum anti-HCV levels were both age-related, in which patients over 40 years of age had a significantly higher anti-HCV positive rate than those younger than 40 years. Among the anti-HCV-positive patients, the average HCV RNA positive rate was 51.66 and 35.93% in males and females, respectively. Spearman rank analysis showed a significantly positive correlation between serum HCV RNA positivity and the level of anti-HCV. The best cut-off value using serum anti-HCV levels to predict the positivity of HCV RNA was determined to be 9.19 signal-to-cut-off ratio (s/co) in males and 10.18 s/co in females.

**Conclusion:**

The prevalence of anti-HCV in the general population of Liaoning Province was around 1.04%, which was higher than that previously reported from a national survey of HCV infection in China. Approximately 42.9% of the anti-HCV-positive patients also tested positive for HCV RNA. However, the positive correlation between the serum anti-HCV and HCV RNA levels suggests that the positivity of serum HCV RNA can be predicted according to the anti-HCV level in anti-HCV-positive patients, which can improve screening and facilitate timely intervention to prevent the spread of infection.

## Background

Chronic hepatitis C virus (HCV) infection is a high risk factor for the development of chronic hepatitis, liver cirrhosis, and hepatocellular carcinoma [1]. According to the World Health Organization, approximately 185 million people worldwide become infected with HCV, and about 350,000 deaths are caused by HCV infection each year [2, 3]. In 1992, a sero-epidemiological survey of viral hepatitis in China showed that the prevalence of anti-HCV detected in samples was 3.2%, leading to an estimate of approximately 40 million people infected with HCV in China [4]. In 2006, the China Center for Disease Control and Prevention reported that the prevalence of anti-HCV in central China (0.67%) was slightly higher than that in eastern and western China (0.37 and 0.31%, respectively), whereas the prevalence in northern China (0.53%) was significantly higher than that in southern China (0.29%). These data suggested that only 5.6 million people are infected with HCV in China with a prevalence of about 1% [5].

With the successful development of direct antiviral agents (DAAs) for HCV and their excellent clinical therapeutic efficacy, HCV infection has now become a clinically curable disease [6–8]. Therefore, it is essential to identify patients in need of antiviral treatment in a timely and effective manner to provide proper therapy. Such effective screening and intervention can not only help to eliminate the active replication of HCV and prevent disease progression, but could also reduce the overall rate of infection and prevalence of HCV in the population by minimizing the chance of transmission [9, 10].

Although the majority of investigations on the prevalence of HCV infection are based on a positive result for serum antibodies against the virus (anti-HCV) [4, 5, 11, 12], the main criterion to provide antiviral treatment for HCV infection clinically is the detection of HCV RNA. Therefore, it is necessary to further evaluate the prevalence of HCV RNA to reassess the population that is most in need of antiviral treatment on the basis of the current epidemiological survey of HCV infection.

To improve the screening of HCV infection, serum anti-HCV examination was required in outpatients scheduled to undergo invasive examinations and/or treatments, along with patients at the emergency department and all inpatient departments of Shengjing Hospital of China Medical University (hereafter referred to as Shengjing Hospital), which is the largest public hospital in Shenyang and has been one of the top 20 largest public hospitals in China since 2016. Individuals that tested positive for anti-HCV were automatically informed through the clinical warning system based on the hospital information system (HIS), and the patients were recruited for further HCV RNA detection.

Clinically, we found significant differences in serum anti-HCV levels among the patients, and patients with low anti-HCV levels tended to be negative for serum HCV RNA. This general finding led us to wonder about the relationship between the serum anti-HCV level and the positivity of HCV RNA. Therefore, we sought to explore whether we could predict the status of serum HCV RNA according to the result of anti-HCV testing. A review of the relevant literature showed that there have been few systematic comparisons and assessments of the correlation between serum anti-HCV level and HCV RNA [13, 14]. However, all of these studies only focused on the level of anti HCV in viremia-positive and negative patients. Moreover, all of these studies were conducted outside China, and thus clinical data for Chinese patients have not been reported. Therefore, we conducted the present retrospective analysis to evaluate the prevalence of anti-HCV and HCV RNA among patients who were tested for serum anti-HCV production at Shengjing Hospital in 2018 with follow-up for HCV RNA detection.

## Materials and methods

### Patients

A total of 192,202 patients who received serum anti-HCV testing at Shengjing Hospital from January 1, 2018 to December 31, 2018, including 77,500 males and 114,702 females, were included in this study. The anti-HCV results were obtained from the database of the laboratory information system (LIS) of Shengjing Hospital. All the patients had no history of hepatitis B virus (HBV) and human immunodeficiency virus (HIV) infections. The patients were divided into two groups based on the date of anti-HCV examination: the training group included patients who underwent an anti-HCV examination from January 1, 2018 to August 31, 2018, whereas the validation group included patients who underwent anti-HCV examination from September 1, 2018 to December 31, 2018 [15]. We further obtained the corresponding HCV RNA results from patients who tested positive for serum anti-HCV from the LIS system of Shengjing Hospital.

### Laboratory tests

The serum anti-HCV and HCV RNA examinations were performed at the Department of Clinical Laboratory of Shengjing Hospital. If the patients had multiple positive results for anti-HCV and/or HCV RNA, we selected the first result for analysis. Anti-HCV was tested using a chemiluminescence assay (Architect; Abbott Laboratories, Abbott Park, IL, USA) according to the manufacturer’s recommendations. An anti-HCV level > 1.0 signal-to cut-off ratio (s/co) was regarded as a positive result. HCV RNA detection was performed on a fully automated Roche COBAS AmpliPrep (CAP) instrument, directly docked to the Roche COBAS TaqMan (CTM) 96 Analyzer. Serum samples were separated from the whole blood by centrifugation, and 650 μl of serum was used to detect the presence of HCV RNA by an automated real-time reverse transcription-polymerase chain reaction (rRT-PCR) instrument according to the manufacturer’s instructions. The rRT-PCR data were analyzed with Ampli link software, version 3.3 [16].

### Statistical analysis

Statistical analysis was conducted with the SPSS24.0 software package (SPSS, Inc., Chicago, IL, USA). Data are presented as the median (minimum, maximum) unless otherwise stated. Continuous variables were compared with the Student t-test when appropriate, whereas categorical variables are presented as percentages, and were compared using the chi-squared test. The Spearman rank test was used for evaluation of the correlation between the variables. The diagnostic capability of anti-HCV in identifying patients with positive HCV RNA was assessed using receiver operating characteristic (ROC) curves. The area under the ROC curves (AUC) was calculated, and the statistical significance of the difference from an AUC value of 0.5 was determined. The optimum cut-off levels for anti-HCV were then obtained. We calculated the sensitivity, specificity, predictive values (both positive and negative), and the Youden’s index of different levels of anti-HCV.*P* < 0.05 was considered statistically significant.

## Results

### Prevalence of anti-HCV in the patient population

Of the 77,500 male and 114,702 female patients who were tested for serum anti-HCV, 940 (1.21%) and 1066 (0.93%) were anti-HCV-positive, respectively. The anti-HCV-positive rate was significantly higher in males than in females (*P* < 0.001, χ^2^ = 36.00). The anti-HCV level was age-related (*P* = 0.003, χ^2^ = 19.961 for males and *P* = 0.015, χ^2^ = 15.722 for females). However, there was no significant differences in the anti-HCV level between males and females(*P* = 0.225,χ^2^ = 1.471).

The positive rate of anti-HCV also differed significantly according to age group, ranging from 0.18 to 2.40% in males and from 0.20 to 2.07% in females, with a general increase in positivity with increasing age (*P* < 0.001,χ^2^ = 601.419 for males and *P* < 0.001,χ^2^ = 513.965 for females). The positive rate of anti-HCV in patients older than 40 years of age was significantly higher than that in patients younger than 40 years in both males and females (*P* < 0.001,χ^2^ = 554.605 for males and *P* < 0.001,χ^2^ = 422.166 for females). Patients older than 60 years showed the highest anti-HCV positive rate, whereas patients younger than 20 years showed the lowest positive rate. However, there was no difference in anti-HCV positive rates between males and females in patients younger than 20 years (*P* = 0.144, χ2 = 2.134), whereas among patients older than 20 years, males had a higher positive rate than females (*P* < 0.001,χ^2^ = 130.228), especially in those older than 40 years (*P* < 0.001,χ^2^ = 36.495) (Table 1).

**Table 1:** Prevalence of anti-HCV and anti-HCV levels in male and female patients of different age groups

	Males			Females		
Age (years)	Anti-HCV (+/−)	Anti-HCV positive rate (%)	HCV-Ab level (s/co)	Anti-HCV (+/−)	Anti-HCV positive rate (%)	HCV-Ab level (s/co)
0–10	43/24,058	0.18	1.93 (1.34, 5.40)	32/16,008	0.20	1.79 (1.39, 2.63)
11–20	5/3429	0.15	2.4 (1.53, 8.57)	13/3038	0.43	1.86 (1.24, 4.89)
21–30	43/5343	0.8	6.37 (1.99, 14.57)	117/22,772	0.51	2.31 (1.43, 8.09)
31–40	60/8952	0.67	3.85 (1.68, 11.86)	147/26,361	0.55	2.65 (1.42, 9.22)
41–50	118/6964	1.67	12.55 (6.86, 14.30)	161/14,170	1.12	7.91 (1.95, 13.24)
51–60	252/10,764	2.29	11.56 (4.44, 14.09)	228/13,847	1.62	10.41 (2.70, 13.67)
61–	419/17,050	2.4	11.99 (3.00, 14.08)	368/17,440	2.07	9.35 (2.57, 13.37)
Total	940/76,560	1.21	11.3 (2.67,14.02)	1066/11,3636	0.93	5.53 (1.87,13.09)

### Prevalence of HCV RNA in anti-HCV-positive patients

Among the total 2006 anti-HCV-positive patients, 1289 (64.26%) returned for a serum HCV RNA examination, the HCV RNA-positive rates is 42.9%. The average HCV RNA-positive rates in male and female anti-HCV-positive patients were 51.66 and 35.93%, respectively, representing a significant difference (*P* < 0.001, χ^2^ = 32.129). Consistent with the results for the serum anti-HCV level, the positive rate of HCV RNA was also age-related (*P* < 0.001, χ^2^ = 101.939) (Table 2), in which the positive rate was significantly higher in patients over 40 years old than in those under 40 years old regardless of gender (*P* < 0.001, χ^2^ = 93.929).

**Table 2:** HCV RNA detection rate and positive rate in the anti-HCV-positive population of different ages

Age (years)	Males				Females			
	Tested for HCV RNA/anti-HCV- positive	HCV RNA test rate (%)	HCV RNA (+)/tested for HCV RNA	HCV RNA positive rate (%)	Tested for HCV RNA/anti-HCV-positive	HCV RNA test rate (%)	HCV RNA (+)/tested for HCV RNA	HCV RNA positive rate (%)
0–10	28/43	65.12	2/28	7.14	24/32	75	0/24	0
11–20	5/5	100	1/5	20	12/13	92.31	1/12	8.33
21–30	33/43	76.74	12/33	36.36	89/117	76.07	17/89	19.1
31–40	41/60	68.33	14/41	34.15	94/147	63.95	18/94	19.15
41–50	65/118	55.08	45/65	69.23	108/161	67.08	42/108	38.89
51–60	168/252	66.67	98/168	58.33	165/228	72.37	76/165	46.06
61–	231/419	55.13	123/231	53.25	226/368	61.41	104/226	46.02
Total	571/940	60.74	295/571	51.66	718/1066	67.35	258/718	35.93

### Correlation between anti-HCV level and HCV RNA

The positive rate of HCV RNA differed significantly according to the anti-HCV levels (*P* < 0.001, χ^2^ = 911.924). The correlation between the serum anti-HCV level and HCV RNA positivity was evaluated for 1289 patients with available corresponding results. The Spearman rank test showed a positive correlation between serum HCV RNA positivity and the level of anti-HCV (*P* < 0.001, r = 0.784; Table 3). Specifically, when the anti-HCV level was lower than 3 s/co, the positive rate of HCV RNA was rarely low, whereas when the anti-HCV level was between 3 and 8 s/co, the positive rate of serum HCV RNA gradually increased with the increase of anti-HCV level, although the HCV RNA-positive rate was still lower than 20%. However, when the anti-HCV level was higher than 8 s/co, the positive rate of serum HCV RNA significantly increased with increasing antibody levels.

**Table 3:** Correlation between serum anti-HCV level and HCV RNA

Anti-HCV level	Tested for HCV RNA/anti-HCV (+)	HCV RNA test rate (%)	HCV RNA (+)/tested for HCV RNA	HCV RNA (+) rate (%)
(1, 2)	294/459	64.05	0/294	0.00
(2, 3)	135/194	69.59	0/135	0.00
(3, 4)	80/113	70.8	2/80	2.50
(4, 5)	50/67	74.63	1/50	2.00
(5, 6)	34/49	69.39	1/34	2.94
(6, 7)	33/51	64.71	3/33	9.09
(7, 8)	25/38	65.79	4/25	16.00
(8, 9)	23/32	71.88	10/23	43.48
(9, 10)	35/54	64.82	20/35	57.14
(10, 11)	45/72	62.50	35/45	77.78
(11, 12)	58/101	57.43	50/58	86.21
(12, 13)	89/155	57.42	78/89	87.64
(13, 14)	131/217	60.37	116/131	88.55
(14, 15)	135/220	61.36	122/135	90.37
(15, 16)	79/119	66.39	71/79	89.87
(16, 17)	23/35	65.71	21/23	91.30
(17, 18)	13/19	68.42	13/13	100.00
(18, ∞)	7/11	63.64	6/7	85.71
Total	1289/2006	64.26	553/1289	42.90

Spearman rank test, r = 0.784, *P* < 0.001

To find the best cut-off value of serum anti-HCV levels to predict the positivity of HCV RNA, the 1289 patients were divided into the training group (897 patients, 396 males and 501 females who were tested for anti-HCV before August 31, 2018) and the validation group (392 patients, 175 males and 217 females who were tested for anti-HCV after September 1, 2018). There were no significant differences in gender, age, anti-HCV level, HCV RNA detection rate, and HCV RNA-positive rate between the two groups (all *P* > 0.05; Table 4).

**Table 4:** Demographic characteristics of patients in the training and validation groups

	Training group	Validation group	*P* (χ^2^ value)
Number	897	392	
Age (years)	55 (40.5, 65)	53 (40, 63)	0.847 (0.037)
Sex (male/female)	396/501	175/217	0.869 (0.027)
HCV RNA (+/−)	383/514	170/222	0.823 (0.05)
Anti-HCV level	8.11 (2.21, 13.645)	6.69 (2.02, 13.22)	0.796 (0.067)
Tested for HCV RNA	897/1382	392/624	0.367 (0.814)

### Diagnostic value of serum anti-HCV level for predicting positive HCV RNA in the training group

**Fig. 1:**
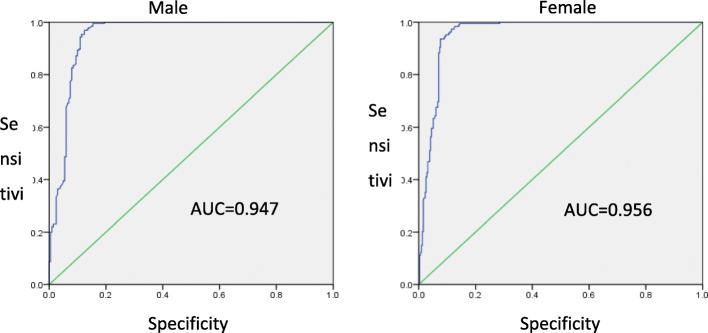
Receiver operating curves (ROC) for the ability of the anti-HCV level to predict the HCV RNA-positive status of male and female patients in the training group

The relationship between serum anti-HCV level and HCV RNA was analyzed according to the ROC curve in the training group. When cut-off anti-HCV levels of 9.19 s/co and 10.18 s/co were used for male and female patients, respectively, the sensitivity was 0.969 and 0.936, and the specificity was 0.876 and 0.923, respectively. The positive predictive value of HCV RNA positivity in males and females was 0.883 and 0.880, respectively, and the negative predictive value was 0.967 and 0.960 in males and females, respectively. The AUC value was 0.947 (0.923–0.971) for male patients and 0.956 (0.939–0.974) for female patients (Fig. 1).

### Predictive ability of serum anti-HCV for HCV RNA in the validation group

Application of the cut-off values obtained from the training group to the validation group showed sensitivity of 0.960 and 0.857, and specificity of 0.867 and 0.952 in male and female patients, respectively. The positive predictive value was 0.906 and 0.896, the negative predictive value was 0.942 and 0.933, and Youden’s index was 0.827 and 0.809 in males and females, respectively, which indicated a good predictive effect with these determined cut-off values of anti-HCV.

## Discussion

Chronic hepatitis C has become a clinically curable disease with the successful application of DAAs, offering the potential for the eradication of HCV. However, effective screening and timely treatment of patients with HCV infection play key roles in controlling the prevalence of HCV infection. In particular, appropriate and accurate screening methods are important during this process [17, 18].

Both serum anti-HCV and HCV RNA are common indicators for HCV infection. Anti-HCV detection is sensitive and convenient, and is therefore often used for screening of HCV infection, whereas HCV RNA detection is considered the “gold standard” for the clinical diagnosis of HCV infection and is the main criterion for antiviral therapy [9]. A positive result for anti-HCV will activate the screening and warning feedback network for HCV infection. That is, the system will remind the doctor to carry out a follow-up HCV RNA examination for anti-HCV-positive patients or to transfer the patients to the department of infectious disease for further evaluation. Based on this system, the anti-HCV screening rate of inpatients and outpatients has reached up to 68.06 and 0.94%, respectively, at Shengjing Hospital since 2016 [19]. Despite this relatively low screening rate for outpatients, majority of the population of childbearing age has undergone this test. Our results showed that more than 60% of anti-HCV-positive patients also underwent HCV RNA testing, making it possible to investigate the correlation between these results.

The Shengjing hospital includes all major clinical departments along with a large number of pediatric patients. The majority of patients reside in Liaoning Province and surrounding areas. Therefore, although our data were obtained from the HIS system of a single institute, the sample can provide a good representation of the prevalence of HCV infection in Liaoning Province. Overall, we found an anti-HCV-positive rate of 1.04%, which is higher than the rate of 0.53% reported previously in northern China in 2006 [4, 5]. We speculate that this increased anti-HCV-positive rate may not reflect an actual increase in the incidence of HCV infection in recent years, but is rather most likely related to the increased awareness of the importance of screening for HCV infection along with the increased sensitivity of anti-HCV detection methods. This is supported by the fact that the anti-HCV-positive population was mainly over 40 years old, and the prevalence was quite low in younger patients in the current study, consistent with previous reports [20, 21].

In line with previous reports showing a difference in anti-HCV-positive rates between sexes [22–25], the positive rates of both serum anti-HCV and HCV RNA in male patients were significantly higher than those in female patients in the current study. Although the precise reasons for this difference in prevalence of HCV infection according to sex are not yet clear, it may be related to different life styles, such as male homosexuality, sharing of equipment used for drug injection, and tattoo, and thus men may have a higher chance of being infected by HCV [26].

The prevalence of HCV infection among different age groups has rarely been reported. Here, we showed a significant difference in the serum anti-HCV-positive rate among different age groups, which increased with increasing age and was significantly higher in patients older than 40 years than in those below 40 years old. This result is consistent with previous studies [18, 27, 28] indicating that the infection rate of HCV in younger people is reducing, which is attributed to the increasingly more strict and standardized inspection and management of blood and blood products [29]. Moreover, there was an interaction between age and sex in that there was no sex difference in anti-HCV-positive prevalence among patients younger than 20 years, whereas males had a higher prevalence in patients older than 20 years. This result further indicated that the difference in the HCV infection rate might not be related to sex itself, but rather to the fact that men have a higher chance of infection due to unhealthy behavior.

Only patients positive for HCV RNA require antiviral therapy; however, not all patients that are positive for anti-HCV harbor detectable levels of HCV RNA in the serum. Nevertheless, we found a significant correlation between the serum anti-HCV level and HCV RNA positivity; the positive rate of HCV RNA was significantly lower in patients with a low anti-HCV level than in patients with a high anti-HCV level. When all of the anti-HCV-positive patients were stratified for age and sex, the positive rate of serum HCV RNA was consistent with the prevalence of anti-HCV overall and within different age groups. HCV RNA positivity also gradually increased with age among the anti-HCV-positive patients, at below 20% in patients under 40 years old but more than 50% in patients over 40 years old. This result further suggested that patients over 40 years are the main target population for HCV infection and treatment.

In the training group, we determined the cut-off values of anti-HCV levels for predicting a serum HCV RNA-positive status at 9.19 s/co and 10.18 s/co in male and female patients, respectively, with high AUC values, sensitivity, and specificity, indicating the good predictive value of the anti-HCV level for serum HCV RNA positivity. These results were confirmed in the validation group. Serum anti-HCV detection is widely used for screening HCV infection worldwide owing to the simple and easy process, with relatively high sensitivity, availability of standardized commercial kits, and no requirement of specialized experimental instruments. Based on our results, serum HCV RNA positivity can be accurately predicted according to the simple measurement of anti-HCV levels in most anti-HCV-positive patients, which can facilitate timely screening and intervention in contexts where HCV RNA detection is inconvenient or unavailable.

The main advantage of this study is that we provide data based on a rare systematic analysis of the prevalence of anti-HCV and HCV RNA in the population of Liaoning Province in the northeast of China. At present, the prevalence of HCV infection reported in the literature is mostly based on anti-HCV detection [4, 5, 11, 12]. We found that Only 42.9% of all anti-HCV positive patients were positive for HCV RNA, indicating that more than half of the anti-HCV-positive patients do not need antiviral therapy; thus, the number of patients who actually require antiviral therapy may have been overestimated previously [30]. This study further revealed a correlation between serum anti-HCV levels and HCV RNA, with a lower positive rate of HCV RNA in patients with a low level of serum anti-HCV. This correlation could offer a solution to practical problems in clinical laboratories to improve the screening and identification of chronic hepatitis C patients.

However, there are still some shortcomings of this study. First, the patient information was obtained from the records of a general hospital. Although the patients are widely distributed across the region, they still cannot fully represent the characteristics of the local population. Moreover, our data were derived from a single hospital excluding data from other hospitals in the region, which could introduce bias. In addition, this study employed a cross-sectional design, and the proportion of female patients was relatively higher, which may have impacted the results.

## Conclusion

Taken together, the prevalence of anti-HCV in Liaoning Province was found to be around 1.04%, which was higher than that reported previously from a national survey of HCV infection [5, 11, 31]. However, less than half of the anti-HCV-positive patients were HCV RNA-positive, which indicted that using anti-HCV as the indicator of HCV infection may result in overestimation of the number of patients requiring antiviral therapy. Nevertheless, the positive correlation between the serum anti-HCV level and HCV RNA suggests that HCV RNA positivity can be predicted according to the specific serum anti-HCV level based on our identified cut-off values, and should be separately considered according to gender.

## Data Availability

The datasets used and analyzed during the current study are included within this article.

## Abbreviations

HBV: Hepatitis B virus
HCV: Hepatitis C virus
anti-HCV: Antibodies against the virus
HIV: Human immunodeficiency virus
CTM: COBAS TaqMan
CAP: COBAS AmpliPrep
DAAs: Direct antiviral agents
rRT-PCR: Automated real-time reverse transcription-polymerase chain reaction
s/co: Signal-to-cut-off ratio
HIS: Hospital information system
LIS: Laboratory information system

## References

[1] Negro F (2014). Epidemiology of hepatitis C in Europe. Dig Liver Dis.

[2] Bai H, Zhang L, Ma L, Dou XG, Feng GH, Zhao GZ (2007). Relationship of hepatitis B virus infection of placental barrier and hepatitis B virus intra-uterine transmission mechanism. World J Gastroenterol.

[3] Mohd Hanafiah K, Groeger J, Flaxman AD, Wiersma ST (2013). Global epidemiology of hepatitis C virus infection: new estimates of age-specific antibody to HCV seroprevalence. Hepatology..

[4] Cui Y, Jia J (2013). Update on epidemiology of hepatitis B and C in China. J Gastroenterol Hepatol.

[5] Chen YS, Li L, Cui FQ, Xing WG, Wang L, Jia ZY (2011). A sero-epidemiological study on hepatitis C in China. Chin J Epidemiol.

[6] Ozaras R, Yemisen M, Balkan İ (2013). Current and future therapies for hepatitis C virus infection. N Engl J Med.

[7] Wei L, Lim SG, Xie Q, Văn KN, Piratvisuth T, Huang Y (2019). Sofosbuvir-velpatasvir for treatment of chronic hepatitis C virus infection in Asia: a single-arm, open-label, phase 3 trial. Lancet Gastroenterol Hepatol.

[8] Zeuzem S, Foster GR, Wang S, Asatryan A, Gane E, Feld JJ (2018). Glecaprevir-Pibrentasvir for 8 or 12 weeks in HCV genotype 1 or 3 infection. N Engl J Med.

[9] EASL (2018). Recommendations on treatment of hepatitis C 2018. J Hepatol.

[10] Bang CS, Song IH (2017). Impact of antiviral therapy on hepatocellular carcinoma and mortality in patients with chronic hepatitis C: systematic review and meta-analysis. BMC Gastroenterol.

[11] Gao Y, Yang J, Sun F, Zhan S, Fang Z, Liu X (2019). Prevalence of anti-HCV antibody among the general population in mainland China between 1991 and 2015: a systematic review and meta-analysis. Open Forum Infect Dis.

[12] Platt L, Easterbrook P, Gower E, McDonald B, Sabin K, McGowan C (2016). Prevalence and burden of HCV co-infection in people living with HIV: a global systematic review and meta-analysis. Lancet Infect Dis.

[13] Ranjbar Kermani F, Sharifi Z, Ferdowsian F, Paz Z, Tavassoli F (2015). The usefulness of anti-HCV signal to cut-off ratio in predicting viremia in anti-HCV in patients with hepatitis C virus infection. Jundishapur JMicrob.

[14] Seo YS, Jung ES, Kim JH, Jung YK, Kim JH, An H (2009). Significance of anti-HCV signal-to-cutoff ratio in predicting hepatitis C viremia. Korean J Intern Med.

[15] Hongbo L, Xiaohui L, Hong K, Wei W, Yong Z (2007). Assessing routine and serum markers of liver fibrosis in CHB patients using parallel and serial interpretation. Clin Biochem.

[16] Johnson K, Green PK, Ioannou GN (2017). Implications of HCV RNA level at week 4 of direct antiviral treatments for hepatitis C. J Viral Hepat.

[17] Duan Z, Jia JD, Hou J, Lou L, Tobias H, Xu XY (2014). Current challenges and the management of chronic hepatitis C in mainland China. J Clin Gastroenterol.

[18] Chlibek R, Smetana J, Sosovickova R, Gal P, Dite P, Stepanova V (2017). Prevalence of hepatitis C virus in adult population in the Czech Republic - time for birth cohort screening. PLoS One.

[19] Wang F, Lu Y, Zhang CP (2018). Construction and application of hepatitis C screening and management information platform. Modern Hospital Manage.

[20] Bennett H, Waser N, Johnston K, Kao JH, Lim YS, Duan ZP (2015). A review of the burden of hepatitis C virus infection in China, Japan, South Korea and Taiwan. Hepatol Int.

[21] Maaroufi A, Vince A, Himatt SM, Mohamed R, Fung J, Opare-Sem O (2017). Historical epidemiology of hepatitis C virus in select countries-volume 4. J Viral Hepat.

[22] Freeman AJ, Dore GJ, Law MG, Thorpe M, Von Overbeck J, Lloyd AR (2001). Estimating progression to cirrhosis in chronic hepatitis C virus infection. Hepatology..

[23] Li JF, Liu S, Ren F, Liu M, Wu HL, Chen Y (2014). Fibrosis progression in interferon treatment-naive Chinese plasma donors with chronic hepatitis C for 20 years: a cohort study. Int J InfectDis.

[24] Rao HY, Sun DG, Yang RF, Liu F, Wang J, Feng B (2012). Outcome of hepatitis C virus infection in Chinese paid plasma donors: a 12-19-year cohort study. J Gastroenterol Hepatol.

[25] Cornberg M, Razavi HA, Alberti A, Bernasconi E, Buti M, Cooper C (2011). A systematic review of hepatitis C virus epidemiology in Europe, Canada and Israel. Liver Int.

[26] Rao H, Xie Q, Shang J, Gao Z, Chen H, Sun Y, et al. Real-world clinical outcomes among individuals with chronic HCV infection in China: CCgenos study. Antivir Ther. 2019. 10.3851/IMP3334. 10.3851/IMP3334 31566575

[27] Zhang M, Wu R, Xu H, Uhanova J, Gish R, Wen X (2019). Changing incidence of reported viral hepatitis in China from 2004 to 2016: an observational study. BMJ Open.

[28] Chen CH, Yang PM, Huang GT, Lee HS, Sung JL, Sheu JC (2007). Estimation of seroprevalence of hepatitis B virus and hepatitis C virus in Taiwan from a large-scale survey of free hepatitis screening participants. J Formos Med Assoc.

[29] Fu P, Lv Y, Zhang H, Liu C, Wen X, Ma H (2019). Hepatitis C virus prevalence and incidence estimates among Chinese blood donors. Transfusion..

[30] Gower E, Estes C, Blach S, Razavi-Shearer K, Razavi H (2014). Global epidemiology and genotype distribution of the hepatitis C virus infection. J Hepatol.

[31] Polaris Observatory HCV Collaborators. Global prevalence and genotype distribution of hepatitis C virus infection in 2015: a modelling study. Lancet Gastroenterol Hepatol. 2017;2(3):161–76. 10.1016/S2468-1253(16)30181-9 28404132

